# Work Related Stress, Well-Being and Cardiovascular Risk among Flight Logistic Workers: An Observational Study

**DOI:** 10.3390/ijerph15091952

**Published:** 2018-09-07

**Authors:** Luigi Isaia Lecca, Marcello Campagna, Igor Portoghese, Maura Galletta, Nicola Mucci, Michele Meloni, Pierluigi Cocco

**Affiliations:** 1 Department of Medical Sciences and Public Health, University of Cagliari, Asse Didattico E, SS 554, km 4,500, 09042 Monserrato, Italy; mam.campagna@gmail.com (M.C.); igor.portoghese@gmail.com (I.P.); maura.galletta@gmail.com (M.G.); pcocco@unica.it (P.C.); 2 Department of Clinical and Experimental Medicine, University of Florence, 50134 Florence, Italy; nicola.mucci@unifi.it; 3 CENTRALABS, University of Cagliari, Engineering Labs, SS 554, km 4,500, 09042 Monserrato, Italy; mikke.049@gmail.com

**Keywords:** work-related stress, cardiovascular risk score, Karasek taxonomy, general well-being, tailored workplace health promotion

## Abstract

Work-related stress is a known occupational hazard, with a putative role on the development of cardiovascular diseases (CVD). Although several investigations have explored the association in various workplace scenarios, none have focused on the airport flight logistic support personnel, a transportation business of crucial importance, potentially exposed to job stress and consequently to an increase in CVD risk. We explored the relationship between work-related stress and cardiovascular risk in 568 healthy workers of a flight logistic support company using the Health and Safety Executive questionnaire, the Framingham Heart Study General Cardiovascular Disease (CVD) Risk Prediction Score, and the WHO general well-being index (WHO-5). We used univariate and multivariate statistical methods to take account of possible confounders. Our results show that a low job support significantly increases the CVD risk score and decreases the WHO well-being index with reference to subjects reporting high support on the job. In addition, the well-being index of workers with high strain jobs appears lower in respect to workers employed in low strain job. The multivariate analysis confirms a protective effect of job support, and shows a detrimental influence on CVD risk by physical inactivity, regular intake of alcohol, and a low educational level. In addition, job control, job support, low strain, and high demand coupled with high control (active job) showed a beneficial effect on psychological well-being. Our results suggest that a combination of general risk factors and organizational factors contributes to increase CVD risk and well-being, representing a crucial target for intervention strategies to promote health in the workplace.

## 1. Introduction

Cardiovascular diseases (CVD) in the adulthood result from the concurrent action of modifiable and unmodifiable risk factors, and represent the major cause of death in industrialized countries [1]. Among the modifiable CVD determinants, tobacco smoking, unhealthy dietary habits, poor physical activity, and stress are known to play a significant role [2]. However, although international consensus exists about work-related stress as one of the major health and safety challenges for modern society [3], its link to cardiovascular risk is still unclear [4,5].

Considering the progressive aging of the world population [6], and its special relevance in some jobs [7], in the near future, a foreseeable increasing proportion of workers might develop chronic diseases during their working life, which would seriously affect services that are crucial for the general population [8].

In recent years, health promotion at the workplace has become an important topic in the international context [9,10]. In the Italian scenario, a recent Memorandum of Understanding between the Italian Society of Occupational Medicine (SIML) and the Italian Ministry of Health highlights the role of occupational physicians in implementing strategies to prevent chronic non-transmissible diseases among workers and citizens, through screening activities and promotion of healthier lifestyles [11]. For these strategies to be effective, a preliminary exploration of the relationships among organizational factors, psychological well-being, and general health status in diverse workplace scenarios would help identify susceptible groups of workers for whom specific workplace health promotion programs may be designed. Despite the association between work-related stress and cardiovascular risk factors, such as metabolic syndrome or hypertension, being well established [12,13], very few studies have explored the association by specific occupation [14,15], and none, as far as we are aware of, has considered occupation in the logistics businesses, such as flight control, safety in communications, maintenance work and security staff working in airports.

The airport flight support business represents a complex workplace, where various job tasks operate in a high security area. The proper management of airport traffic requires an adequate level of coordination between job tasks, some of which, such as those of flight controllers and security personnel, carry a high level of responsibility in taking quick decisions. Under conditions of higher air traffic, job tasks might become stressful and demanding, while the level of job control remains low. However, studies addressing work-related stress in these workers are lacking, and the analysis of the components within the job-strain model seems particularly complicated in respect to other jobs [16].

Validated methods for the assessment of work-related stress take advantage of self- administered questionnaires, such as the Effort/Reward Imbalance (ERI) [17], and the Job Demand-Control (JDC) questionnaires [18], both acknowledged as highly reliable. Specifically, Karasek (1979) identified job demand and job control as key risk factors for employee’s well-being [19]. In the JDC model, job demand is defined by quantitative aspects, such as workload and time pressure; job control, also termed as “decision latitude”, refers to the workers’ ability to control their work activity, in terms of workload and schedule. In combining the two dimensions of job demand and job control, Karasek classified jobs into four categories: high strain jobs (high on demand and low on control), low strain jobs (low on demand and high on control), passive jobs (low on demands and low on control), and active jobs (high on demands and high on control) [19]. The JDC Model and its jobs taxonomy was extended to job support as a third component [20]. In its further theorization, the JDC Model proposed that job characterized by high demands, low control, and low social support to be most harmful for workers’ well-being [21].

We adopted a cross-sectional study design to assess the relationship between work-related stress, general perception of emotional well-being, and cardiovascular risk among employees of a logistics company for safety in communications and flight. A secondary objective was to identify indicators of effectiveness of health promotion programs targeted on healthy lifestyles, and tailored on this specific organizational context.

## 2. Materials and Methods

### 2.1. Study Population

During the 2016–2017 annual workplace health surveillance program, we recruited 617 workers of a logistic company supporting safety in communications and flight, out of an overall workforce of more than 1000 workers. Eligible subjects (*n* = 617) were not randomly selected, but they were those scheduled for undergoing the workplace health surveillance program in the study period. The inclusion criteria were: being in service on 1 January 2014 or earlier, and being aged 30–74 years. Females were excluded being too few for any assessment of gender-related differences. Following the exclusions, 568 subjects were retained for the analysis. Table 1 shows the exclusion criteria and the number of subjects excluded by cause. The acceptance rate for questionnaire was 99.5%.

During the clinical exam, the eligible subjects were informed about the purposes of the study and asked to provide written consent to participate. The company operates in two low traffic airports and two training areas in Italy, with flight schedules only in established periods along the year (training periods). Workers were engaged in three shift-work schedules: daytime fixed work shifts (reference, administrative tasks and flight control personnel who operate only in office settings); 12 h work shifts (*h*12, technical support personnel such as electricians, mechanics, drivers, radar and communication operators, and firefighters, with a Day–Night–Rest–Rest–Day–Night schedule (DNRRDN)); and 24 h work shifts followed by 96 h rest (*h*24, security personnel).

For each study subject, we abstracted the following data from the medical records: age, educational level, job tasks, job seniority, work shifts, body mass index (BMI), heart rate at rest, systolic and diastolic blood pressure, health history, and current medical treatments. A trained physician measured systolic (SBP) and diastolic blood pressure (DBP) during the clinical exam, with a manual sphygmomanometer, after two minutes in the supine position. We considered overweight subjects with a BMI exceeding 25 kg/m^2^, and we set the threshold for hypertension at SBP ≥ 140 mmHg or DBP ≥ 90 mmHg [22,23]. Each subject filled a self-administered standard questionnaire to assess work-related stress, which included also information on lifestyle habits, such as smoking, alcohol intake, recreational physical activity, and coffee intake.

We categorized lifestyle, personal, and occupational variables as it follows:

- smoking habit: current smokers vs. former smoker or never smokers;
- recreational physical activity: regular, if the subject exercised at least twice per week vs. sporadic (<2 times per week) or none;
- alcohol intake: abstinent or sporadic (<1 alcohol unit per day, such as social drinking) vs. only on the weekend vs. at least 1 alcohol unit daily;
- coffee intake: low (<2 per day), medium (2–4 per day), and high (>4 per day);
- type of shiftwork schedule: fixed daytime workers vs. h12 vs. h24 shift workers;
- education: low (≤8 years of education), medium (8–13 years), and high (>13 years with a degree); and
- job tasks: operative jobs, security personnel, administrative jobs/ flight control personnel.

### 2.2. Work Related Stress and Well-Being Assessment

All the participants filled a self-administered questionnaire including the Italian version of UK Health and Safety Executive (HSE) Management Standards Indicator Tool [24], with the subscales for job demand (8 items), job control (6 items), and support from coworkers (horizontal support; 4 items) [25]. The internal reliability for each subscale, expressed by the Cronbach’s α values, was 0.84, 0.80 and 0.83, respectively.

Based on the Karasek’s job taxonomy [18], we defined passive jobs (jobs low on demands and low on job control), low strain jobs (jobs low on demands and high on job control), high-strain jobs (jobs high on demands and low on control) and active jobs (high on job demands and high on job control) by combining job demand and job control scores according to whether they were below or above the median in a two-by-two combination matrix. We also stratified the four categories of Karasek’s job taxonomy by high vs. low job support from peers, defined by the job support score above or below the median, respectively [20].

We used the WHO-Five Well-Being Index (WHO-5) to measure well-being in study participants; the WHO-5 Index is composed of 5 items [26]: (1) feeling cheerful and in good spirits; (2) feeling calm and relaxed; (3) to feel active and vigorous; (4) waking up feeling fresh and rested; and (5) having a daily life filled with interesting things. Items were answered on a 5-point scale from 1 (strongly disagree) to 5 (strongly agree), representing the perceived frequency of each item in the last two weeks. The raw score was expressed by the sum of the individual five scores. The raw score therefore theoretically ranges from 0 (absence of well-being) to 25 (maximal well-being) [26]. The internal reliability for this subscale, calculated by the Cronbach’s α, was 0.88.

### 2.3. Cardiovascular Risk Assessment

For each study subject, we calculated the Framingham Heart Study General Cardiovascular Disease (CVD) Risk Prediction Score [27], which represents the absolute CVD risk as the percent probability of developing a major CVD within ten years, considering concurrent risk factors, such as age, diabetes, smoking, treated or untreated hypertension, serum total and HDL cholesterol, or BMI as an alternative to blood lipids. We considered the following as major CVD events: coronary death, myocardial infarction, coronary insufficiency, angina, ischemic stroke, hemorrhagic stroke, transient ischemic attack, peripheral artery disease, and heart failure. This model is applicable to individuals aged 30–74 years, free from a CVD diagnosis at the baseline clinical exam [28]. As blood lipid level was not included in the routine workplace health surveillance, we used BMI instead, as suggested for the simpler Framingham Study Risk Prediction Score model. CVD risk was categorized as low (<10%), moderate (10–20 %), or high (>20%).

### 2.4. Statistical Analysis

We used descriptive parametric and nonparametric statistics as appropriate, and we tested the differences by study groups with the analysis of variance (ANOVA) or the Kruskal–Wallis test. Based on the univariate correlation matrix among continuous and categorical variables, we modeled CVD risk as a function of work-related stress (scores of job demand, job control, and job support), adjusting for education, alcohol intake, recreational physical activity, and DBP with multiple regression analysis. We also set a linear regression model predicting well-being index as a function of the work-related stress scores adjusting for age, duration of employment, educational level, alcohol intake, smoking habit, BMI, SBP and DBP. We also set a third regression model to predict CVD risk and WHO-5 well-being index, by replacing the work-related stress scores with the Karasek’s categories and job support categories instead of stress scores CVD risk and well-being, and including shift schedule as an independent explanatory variable. The analysis was conducted with SPSS® v24 (IBM Corp, Armonk, NY, USA).

Due to the observational nature of our study, in absence of any additional procedure beyond the routine mandatory health screening protocol, and in absence of any involvement of therapeutic medication, no formal approval of the Institutional Review Board, the Ethics Committee of the Cagliari University Hospital was required. Nonetheless, all subjects were informed about the study and those enrolled in the study gave written informed consent prior to participation. Questionnaires were anonymous, coded and safely stored by the PI (MC), the only enabled to access, separately from the clinical documentation of each participant. The study was conducted in accordance with the Helsinki Declaration.

## 3. Results

Table 2 shows selected characteristics of the study population. Mean age of the 568 male participants was 44.9 years (*sd* 6.69), and mean BMI was 25.9 (*sd* 2.92). Current smokers accounted for 23.6% of the study population; overweight subjects were 57%, regular alcohol drinkers 28.9%, and 59.7% practiced regularly recreational physical activity; and 26.6% suffered from hypertension.

Table 3 shows the distribution of study subjects by Karasek’s categories and job support categories. Passive jobs were less represented (16.2%), and the low strain category prevailed (32%). The four Karasek’s categories were subdivided by job support (low: 48%; high: 52%), with cut points of the job demand, control and support scores of 15, 22, and 17, respectively, according to Karasek’s categorization.

Subjects in the four Karasek’s categories and the two job support categories had similar age, duration of employment, BMI, SBP, DBP, and HR (not shown in tables).

Sixty-four percent study subjects were classified at low CVD risk (CVD risk < 10%), about 29% at moderate risk (CVD risk between 10% and 20%) and 7% at high risk (CVD risk > 20%). The median CVD risk of the overall population can be considered as moderate (7.7%), with the individual score range between 1% and 56% [29] (Table 4).

**Figure 1:**
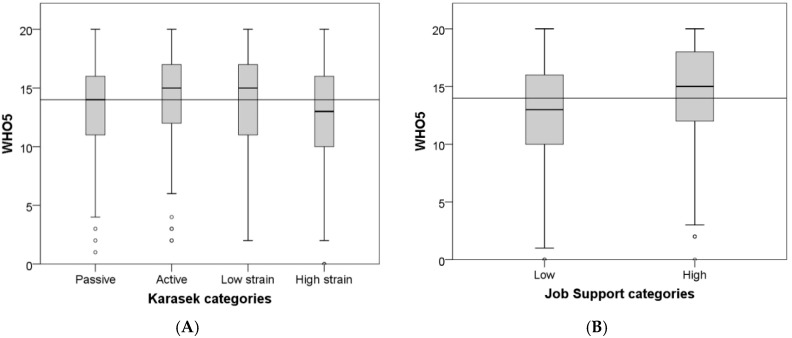
Main median (grey line) and box plots of general well-being (WHO-5): in the four Karasek’s categories (**A**); and in the job support categories (**B**).

We observed lower values of the WHO well-being index in the high strain job category with respect to low strain jobs (*p* = 0.005) (Table 3 and Figure 1A). The mean CVD risk score did not pass the low-level threshold in any of the four Karasek’s categories, and it did not vary among those categories. A low job support, however, moderately though significantly increased CVD risk score, and decreased the WHO well-being index in respect to subjects reporting high support on the job (Table 4 and Figure 1B).

The correlation matrix in Table 5 indicates that the CVD risk score was directly correlated with regular intake of alcoholic drinks (*p* = 0.023) and heart rate at rest (*p* < 0.001), and inversely correlated with recreational physical activity (*p* ≤ 0.001), the well-being index (*p* ≤ 0.001), and the job support score (*p* = 0.037). On the other hand, the WHO-5 well-being index was directly correlated with job control (*p* < 0.001) and job support (*p* < 0.001), and inversely correlated with job demand (*p* = 0.047). DBP was well correlated with SBP (Pearson’s correlation coefficient = 0.702, *p* < 0.001, not shown in the Table 5, and moderately correlated with CVD risk (Table 5).

The linear regression model predicting CVD risk showed a detrimental effect of diastolic blood pressure, low educational level, and physical inactivity, which tended to increase CVD risk. On the contrary, a higher job support score was protective against CVD risk, as well as abstinence from or occasional intake of alcoholic beverages (Table 6). The median values of predicted CVD risk stratified by job support and Karasek’s categories are reported in Table 6.

Including Karasek’s categories, instead of the job demand, control and support scores, in the regression model predicting CVD risk did not result in any significant effect on CVD risk (R^2^ = 0.279; adjusted R^2^ = 0.265). When adjusting results for shiftwork schedule, the diurnal shift schedule showed a protective effect with respect to the h24 shift schedule (regression coefficient = −2.063; se = 0.935; *p* = 0.03).

A high score of job control and job support showed a beneficial effect on well-being, while older age and physical inactivity manifested a detrimental effect (Table 7). When Karasek’s categories and job support categories replaced the scores of their components in the regression model, low strain job and active job showed a significant effect on increasing well-being with respect to high strain job and passive job, while the low job support category showed the opposite effect. Shift work schedule did not show any effect on well-being (Table 8). Median Predicted CVD risk score in the four Karasek’s categories and the two job support categories, based on the linear regression model, are reported in Table 9.

## 4. Discussion

Consistent with previous reports [30], our cross sectional study of workers of a logistic company suggests job support from peers protects against CVD risk, thus confirming the hypothesis that some dimensions of work-related stress affect cardiovascular risk. More specifically, modifiable and unmodifiable risk factors might contribute to increase CVD risk and well-being in workplaces where job support from peers is low [3,31]. Reversely, general well-being and job support reportedly increase long-term survival in chronic heart disease patients [32]. The assessment of work-related stress, based upon the classical dimensions postulated by Karasek, showed a balanced distribution of the study population among the four Karasek’s categories, with high strain jobs accounting for about one fourth of population, similar (22–25%) to what reported in Italian nurses [33]. On the other hand, the median scores of job demand and job control in this flight logistic support company were higher than in similar studies conducted in a mixed population with the same evaluation method (15 vs. 12 for job demand; 22 vs. 18 for job control) [30].

High strain jobs have been repeatedly associated with adverse health outcomes, such as cardiac autonomic imbalance, as suggested by Heart Rate Variability, hypertension, or metabolic syndrome. [33,34,35,36,37]. In our study population, we observed an effect of job strain on perceived well-being, but not on CVD risk. Perhaps, working in a company providing logistic support for safety in communications and flight would not imply physical tasks demanding enough to affect job strain perception.

As it concerns other known CVD risk factors, consistent with previous reports [38], we observed a protective effect of education. Education is frequently used as a surrogate for socioeconomic status in epidemiological studies [39], which in turn implies more frequent involvement in high strain, low control, and low support jobs.

Our results are also consistent with previous reports of a direct influence of work-related stress dimensions on psychological well-being, independent on other possible confounders such shift work [16]. In addition, consistent with other reports [40], in our study, population shift work schedule affected CVD risk, but it was not related to perceived well-being. It is worth noting that the *h*24 shift work schedule was well tolerated by workers participating in our study, thanks to the long recovery time following each work shift. However, other conditions not considered in the present study, such as home stress or factors associated with the specific job task (e.g., security personnel) might have affected our results.

Mean age of our study population was 44.9 years (*sd* 6.7), which suggests a substantial aging of the working population, even in the logistic support business. Aging of the workforce is shared by most developed economies, and it can adversely impact labor productivity [41]; therefore, active aging is a main area of concern in occupational medicine and public health [42,43], and occupational physicians need to consider comorbidities and chronic diseases associated with aging, an unmodifiable CVD risk factor. In this context, occupational physicians could play a crucial role, paying attention to work-related stress as well as to risk factors not necessarily related to workplace exposures, such as unhealthy lifestyles. In our study population, the prevalence of current smokers (23.6%) and overweight people (57%) were above the average in the Italian population (19.3% and 54.8%, respectively) [44]. We also observed a clear protective role of regular recreational physical activity in reducing CVD risk. As such, risk factors are modifiable, implementing health promotion strategies to reduce the prevalence of overweight and smoking in these workers might contribute to improve well-being and productivity. Health promotion strategies, targeted towards physical activity and smoking, have been successfully introduced in several workplaces, resulting in a considerable reduction in CV risk [45,46].

In our study population, the average probability of a major cardiovascular event within 10 years was 7.7%, which implies that about 8% will experience a serious health problem during their working life. Following a major cardiovascular event, the occupational physician has to consider its economic and social consequences for the workers, such as return to work, fitness to work, loss of productivity, and loss of professional skills [47]. Easy tools to calculate CVD risk, such as the one we used in this study, might help occupational physicians to identify, during the periodical workplace health surveillance, otherwise healthy workers who might be susceptible to develop a cardiovascular disease. The periodical workplace health surveillance programs are therefore a privileged observatory for the occupational physician to predict CVD risk in otherwise healthy subjects who do not feel the need to consult a medical specialist.

Limitations affecting interpretation of our findings include the cross sectional study design, which does not allow *per se* conclusive inference; in addition, the complexity of tasks in the flight logistic support business could make it difficult to compare results with other workplaces implying more homogeneous tasks. Thirdly, we could not assess the role of stress and social support at home, which might interact with work-related stress in increasing cardiovascular risk [48,49]. Despite such limitations, an advantage of our study was to focus on the overall health status, both mental and physical, of this working population by combining the assessment of work-related stress with that of CVD risk. By using such a holistic approach, occupational physicians might address modifiable factors, such as work stress management through interventions on work organization, as well as lifestyle factors.

## 5. Conclusions

The present study adds new information about the interrelation between theoretical models predicting CVD risk and early changes in biological parameters, before the development of overt disease. Our results suggest that work related stress dimensions may influence CVD risk and psychological well-being among workers. Job support from peers, the decision latitude, the type of job performed and shift work schedule should be considered when aiming to reduce CVD risk by intervening on organizational stress and by promoting healthy lifestyles. Further investigation with a longitudinal study design is warranted to confirm the hypothesis that specific markers of work-related stress and general well-being predict susceptibility of developing cardiovascular disease.

## Figures and Tables

**Table 1:** Flow chart from eligible subjects to analyzed study population.

	*n.*	%	Denominator
Total workforce	1572	100	1572
Potentially eligible for study	617	39.2	1572
Confirmed eligible	568	92	617
Included in the study	568	92	617
Causes for exclusion			
- Female gender	2	0.3	617
- <30 years of age	39	6	617
- Previous diagnosis of CVD	4	0.6	617
myocardial infarction	2	0.3	617
ischemic heart disease	1	0.1	617
dilated cardiomyopathy	1	0.1	617
- Questionnaire incomplete	3	0.5	617
- Did not match the criteria for CVD risk calculation (SBP < 90 mmHg).	1	0.1	617

**Table 2:** Selected variables on the overall study population. Data were available for all the 568 participants.

Parametric Variables	Min.	Max	Mean	*sd*
Age (years)	30	63	44.9	6.69
Duration of employment (years)	1	39	7.00	7.37
Weight (kg)	57	118	78.9	9.90
Height (m)	1.52	1.92	1.74	0.06
BMI (kg/m^2^)	19.3	39.5	25.9	2.92
SBP (mmHg)	90	180	126.6	13.71
DBP (mmHg)	60	110	80.8	8.24
Heart Rate (bpm)	39	132	66.3	11.57
**Non-Parametric Scores**	**Median**		**Interquartile Range**	
WHO-5 index	14		11–17	
CVD risk score	7.7		5.02–12.47	
Job Demand score	15		12–18	
Job Control score	22		19–25	
Job Support score	17		15–18	
**Categorical Variables**		**N**		**%**
Physical activity	Sporadic/none	229		40.3
	regular	339		59.7
Smoking habit	Never/former	434		76.4
	Smokers	134		23.6
Alcohol intake	abstinent	278		48.9
	weekend	126		22.2
	regular	164		28.9
Coffee	Low	170		29.9
	Medium	315		55.5
	High	83		14.6
Educational level	Low	236		41.5
	Medium	314		55.3
	high	17		3.0
Shiftwork schedule	Fixed diurnal	415		73.1
	*h*12	104		18.3
	*h*24	49		8.6
CVD risk class	Low < 10%	365		64.3
	Medium 10–20%	163		28.7
	High > 20%	40		7
Jobs	Operative jobs	447		78.7
	Security personnel	36		6.3
	Administrative jobs/flight control personnel	82		14.4
	Missing	3		0.5

**Table 3:** Distribution of the overall study population by Karasek’s categories and by Job support categories.

Karasek’s Categories	Low Job Support N. (%)	High Job Support N. (%)	All N. (%)
High strain	100 (36.1)	55 (18.9)	155 (27.3)
Low strain	58 (20.9)	124 (42.6)	182 (32.0)
Passive	51 (18.4)	41 (24.4)	92 (16.2)
Active	68 (24.5)	71 (18.9)	139 (24.5)
All	277 (100)	291 (100)	568 (100)

**Table 4:** Kruskal–Wallis test results comparing CVD risk, WHO-5 among Karasek’s categories and Job support categories (overall study population).

	Passive		Low Strain		Active		High Strain		Kruskal–Wallis
	N = 92 (16.2%)		N = 182 (32%)		N = 139 (24.5%)		N = 155 (27.3%)		*p*
	Med	IQR	Med	IQR	Med	IQR	Med	IQR	
CVD risk score	7.6	4.72–10.82	7.5	5.05–12.52	8.5	5–13.4	8.0	5.2–11.7	0.463
WHO-5	14.0	11–16	15.00	11–17	15.0	12–17	13.0	10–17	0.005
	**Low Support**				**High Support**				***p***
	**N (%) = 277 (48.8)**				**N (%) = 291 (51.2)**				
	**Median**		**IQR**		**Median**		**IQR**		
CVD Risk score	8.4		5.45–12.4		7.3		4.4–12.6		0.042
WHO-5	13		10–16		15		12–18		<0.001

**Table 5:** Spearman correlation matrix (* *p* < 0.05; ** *p* < 0.01).

	CV RISK Score	Educational Level	Duration of Employment	Alcohol	Coffee	Physical Activity	WHO-5	Heart Rate	Demand Score	Control Score	Support Score	DBP
**CVD RISK score**	1.000	−0.286 **	0.061	0.096 *	0.029	−0.183 **	−0.217 **	0.211 **	0.038	0.044	−0.088 *	0.415 **
**Educational level**		1.000	−0.115 **	−0.059	−0.013	0.071	0.047	−0.121 **	−0.014	0.032	0.065	−0.100 *
**Duration of employment**			1.000	0.033	0.016	0.011	0.002	0.024	0.097 *	0.035	0.040	−0.018
**Alcohol**				1.000	0.021	−0.044	−0.043	−0.006	−0.023	−0.002	−0.060	0.071
**Coffee**					1.000	−0.022	−0.047	−0.015	0.002	−0.011	−0.091 *	−0.110 **
**Physical activity**						1.000	0.089 *	−0.253 **	−0.027	−0.021	−0.054	−0.112 **
**WHO-5**							1.000	−0.022	−0.083 *	0.162 **	0.319 **	−0.032
**Heart Rate**								1.000	0.010	−0.045	0.053	0.198 **
**Demand score**									1.000	−0.232 **	−0.235 **	0.071
**Control score**										1.000	0.321 **	−0.021
**Support score**											1.000	−0.051
**DBP**												1.000

**Table 6:** Multiple linear regression predicting CVD risk score (R^2^ = 0.2713; adjusted R^2^ = 0.2595). Independent variables: educational level, alcohol intake, DBP, job demand score (continuous), job control score (continuous), job support score (continuous), and physical activity.

Variables	Regression Coefficient	Standard Error	*p*-Value
Intercept	−12.3432	3.5383	0.0005
Job demand Score	−0.0055	0.0562	0.9218
Job control Score	0.0010	0.0642	0.9871
Job support Score	−0.2003	0.0946	0.0348
Education low	3.0705	1.5274	0.0449
Education medium	−0.1747	1.5076	0.9078
Education high	0		
Alcohol abstinent	−1.5613	0.6012	0.0097
Alcohol weekend	−2.2325	0.7279	0.0023
Alcohol regular	0		
Physical activity no	2.0583	0.5237	<0.0001
Physical activity yes	0		
DBP	0.3051	0.0313	<0.0001

**Table 7:** Multiple linear regression predicting WHO-5 well-being (R^2^ = 0.1661; adjusted R^2^ = 0.1447). Independent variables: job demand score (continuous), job control score (continuous), job support score (continuous), age, duration of employment, BMI, SBP, DBP, educational level, alcohol intake, physical activity, and smoking habit.

Variables	Regression Coefficient	Standard Error	*p*-Value
Intercept	9.227238	2.644922	0.0005
Job demand Score	0.016800	0.036697	0.6473
Job control Score	0.118277	0.041802	0.0048
Job support Score	0.350821	0.061604	<0.0001
Age	−0.144603	0.027782	<0.0001
Duration of employment	−0.018262	0.023119	0.4299
BMI	−0.022687	0.059988	0.7054
SBP	0.008469	0.017112	0.6209
DBP	0.024156	0.028681	0.4000
Education low	0.020035	1.024678	0.9844
Education medium	−0.360648	1.003554	0.7195
Education high	0		
Smoke yes	0.551132	0.407182	0.1764
Smoke no	0		
Alcohol abstinent	0.333081	0.389784	0.3932
Alcohol weekend	0.365157	0.473053	0.4405
Alcohol regular	0		
Physical activity no	−0.702412	0.347177	0.0435
Physical activity yes	0		

**Table 8:** Multiple linear regression predicting WHO-5 well-being (R^2^ = 0.1427; adjusted R^2^ = 0.1159). Independent variables: Karasek’s categories, job support categories, BMI, duration of employment, SBP, DBP, educational level, alcohol intake, physical activity, smoking habit, and shift schedule.

Variables	Regression Coefficient	Standard Error	*p*-Value
Intercept	17.582239	2.460948	<0.0001
Passive job	0.384755	0.531633	0.4695
Low strain job	1.107803	0.451790	0.0145
Active job	1.334118	0.472754	0.0049
High strain job	0		
Low support	−1.752851	0.349728	<0.0001
High support	0		
BMI	0.003551	0.061233	0.9538
Age	−0.154161	0.028269	<0.0001
Duration of employment	−0.015653	0.023584	0.5071
SBP	0.009537	0.017722	0.5907
DBP	0.026729	0.029447	0.3644
Education low	−0.217933	1.044671	0.8348
Education medium	−0.611723	1.023010	0.5501
Education high	0		
Alcohol abstinent	0.422055	0.398017	0.2894
Alcohol weekend	0.573967	0.483081	0.2353
Alcohol regular	0		
Physical activity no	−0.755701	0.354033	0.0332
Physical activity yes	0		
Smoke yes	0.631464	0.413891	0.1277
Smoke no	0		
Daily shift	−0.244642	0.614323	0.6906
H12 shift	−0.157054	0.698115	0.8221
H24 shift	0		

**Table 9:** Median Predicted CVD risk score in the four Karasek’s categories and the two job support categories, based on the linear regression model presented in Table 6.

Karasek’s Categories	Low Job Support Median Predicted CVD Risk (IQR)	High Job Support Median Predicted CVD Risk (IQR)
Passive	10.18 (7.28–11.94)	8.93 (5.78–11.59)
Low strain	9.87 (8.33–12.39)	8.54 (6.59–11.32)
Active	10.11 (7.54–12.47)	9.85 (5.78–11.63)
High strain	10.29 (7.39–12.93)	8.44 (6.85–12.14)
